# Development of a digital platform to improve community response to overdose and prevention among harm reduction organizations

**DOI:** 10.1186/s12954-022-00636-2

**Published:** 2022-06-03

**Authors:** Kasey Claborn, Suzannah Creech, Fiona N. Conway, Nina M. Clinton, Katlyn T. Brinkley, Elizabeth Lippard, Tristan Ramos, Jake Samora, Aaron Miri, Justin Benzer

**Affiliations:** 1 The University of Texas at Austin, Austin, TX USA; 2 Baptist Health, Jacksonville, Florida, USA

## Abstract

The overdose crisis in the USA remains a growing and urgent public health concern. Over 108,000 people died due to overdose during 2021. Fatal and non-fatal overdoses are under-reported in the USA due to current surveillance methods. Systemic gaps in overdose data limit the opportunity for data-driven prevention efforts and resource allocation. This study aims to improve overdose surveillance and community response through developing a digital platform for overdose reporting and response among harm reduction organizations. We used a community-engaged, user-center design research approach. We conducted qualitative interviews with *N* = 44 overdose stakeholders including people who use drugs and harm reductionists. Results highlighted the need for a unified, multilingual reporting system uniquely tailored for harm reduction organizations. Anonymity, data transparency, protection from legal repercussions, data accuracy, and community-branded marketing emerged as key themes for the overdose platform. Emergent themes included the need for real-time data in a dashboard designed for community response and tailored to first responders and harm reduction organizations. This formative study provides the groundwork for improving overdose surveillance and data-driven response through the development of an innovative overdose digital platform.

## Introduction

Overdose remains a serious public health problem in the United States (U.S.). During the COVID-19 pandemic, illicit drug use and fatal overdoses have risen by a troubling degree across the USA, implicating synthetic opiates (e.g., fentanyl, carfentanyl) and psychostimulants as main drivers [1]. Texas has not been spared from this spike in overdoses, with the state seeing an increase of 33.5% in reported drug overdose fatalities from 2019 to 2020 [2]. Texas is the second most populous state in the USA, [3] yet the number of opioid overdose deaths was estimated to be only 1402 in 2018 [4]. Compared to the rate in California of over 2400 in 2018, the most populous state in the USA, these data indicate that Texas does not have a significant overdose problem relative to the rest of the nation [5]. Based on size alone, Texas would be expected to experience a much higher number of overdoses; therefore, researchers, public health experts, community health workers, and harm reductionists are increasingly concerned that overdose data are severely under-reported in Texas [6]. Qualitative data with harm reduction workers indicate that Texas does has a more significant problem than the data represents with harm reduction stakeholders estimating that 50–70% of overdoses are not accounted for in existing public health datasets [7].

Several factors likely account for under-reporting of overdoses in Texas. Texas is unique in that it contains vast rural communities, borders Mexico, and has diverse needs. Texas has 254 counties and only 15 have medical examiners. Consequently, drug overdoses are counted differently across counties with Justices of the Peace recording the cause of death in 239 counties. In Texas, Justices of the Peace are elected officials and are not required to be medically trained; however, during their first year of service, they receive 80 h of training in death investigation and 20 h in each subsequent year [8]. Justices of the Peace often do not conduct toxicology tests when there is a death without an obvious cause and must weigh the costs of obtaining an autopsy ($2,500 plus transportation costs to an urban area) with competing county priorities which may result in misdiagnosis and under-reporting of fatal overdoses statewide [8, 9]. Further, Texas policies do not provide adequate protection for people who report an overdose from legal repercussions resulting in a fear of calling emergency management systems (EMS) or going to the emergency department when an overdose occurs. In September 2021, Texas passed a Good Samaritan Law (H.B. No 1694) allowing bystanders who see someone experiencing an overdose to call emergency services with protection from prosecution [10]; however, this law has significant caveats limiting its protections and effectiveness. Specifically, this law does not protect individuals who have called 911 for an overdose within the past 18 months, those who have been convicted of a felony, or those who have used this same protection when calling for a previous overdose.

Within the State of Texas, there are harm reduction organizations spread across cities and counties. The National Harm Reduction Coalition defines harm reduction as a series of practical strategies and ideas directed at lowering negative consequences related to drug use. Of note, syringe service programs and distribution of fentanyl testing strips are illegal in Texas under the drug paraphernalia law [11, 12]. The city of San Antonio is an exception and legalized syringe service exchange in 2007; however, due to local prosecutorial opposition, the syringe service pilot program did not launch until 2019 [13, 14]. Most of the harm reduction organizations located in Texas are community-based with limited resources. A 2020 report published by the Civil Rights Clinic at The University of Texas School of Law and the Texas Harm Reduction Alliance [15] identified ten community-based organizations delivering evidence-based harm reduction services across the state of Texas. Of note, some of these organizations closed permanently during the COVID-19 pandemic and new organizations are emerging. Many of these organizations have unstable funding structures, are volunteer-run, and operate on donations and grant funding. These programs offer a variety of services such as distribution of blankets, condoms, safe use kits, conduct rapid testing for HIV and Hepatitis C, provide wound care, distribute naloxone, and develop relationships with local police departments to facilitate distribution of fentanyl testing strips and syringe exchange. These programs are located in the following urban areas in Texas: Abilene, Austin, El Paso, Fort Worth, Houston, Midland, San Antonio, and Waco. A vast majority of Texas does not have access to harm reduction programs.

As a result of limited resources, harm reduction organizations in Texas do not possess sophisticated methods for tracking data in terms of number of overdose reversals or number of overdoses occurring in their region. These community-based organizations may be an important avenue toward improving surveillance data as they have established trust among people who use drugs and frequent interaction with the community; consequently, these organizations have the potential to capture data among key target populations that do not come into contact with the healthcare system following an overdose.

Taken together, these factors indicate a dire need for improved overdose surveillance to better inform data-driven decisions for resource distribution and prevention efforts in Texas. Accurate reporting systems are critical for funding allocation and to improve access to substance use treatment, overdose prevention efforts, and harm reduction services. The goal of this project was to employ community-engaged research and user-centered design methods to develop an innovative overdose reporting platform for harm reduction organizations that will improve resource tracking and provide closer to real-time data for overdose to allow data-informed community prevention and response efforts across Texas.

## Methods

This study and all procedures were approved by the [Anonymous] Institutional Review Board.

*Community-engaged research approach to user centered design.* The principles of two theoretical frameworks guided study design (see Table 1). User Centered Design (UCD) principles seek to better align products with the intended needs and desires of the target users, rather than the developers [16, 17]. This framework emphasizes a balance between engineering and design through engaging potential users early and often to result in a useable product, which is the key goal of UCD. At its core, the goal of UCD is to avoid developing a digital solution that is not used because it prioritized the needs of the designer or funder. Thus, from a UCD perspective several key processes are critical for optimizing usability: identification of users, user needs, and user constraints and rapid, iterative prototyping and simplification. These processes were executed through a design sprint process pioneered by Google Ventures which is a methodology used to rapidly validate ideas and concepts for solving challenging problems through solutions mapping, evaluating existing approaches and sketching out new solutions, deciding which sketch to pursue and storyboarding the planned solution, prototyping, and testing with end users [18, 19]. Although this manuscript describes our platform development phase, later phases include pilot testing by users and additional feedback and iteration.

**Table 1:** Methodological approach to technology development

UCD concepts and methods	User-centered design (UCD) principles	Community engagement approaches
Careful identification of users and their needs	The UCD field places strong emphasis on explicitly identifying primary, secondary, and sometimes tertiary users in order to ensure that new products effectively meet their needs	Qualitative Interviews, Community Advisory Boards, networking (primary: Harm Reduction Organization champions; secondary: Harm Reduction Organization employees and volunteers; tertiary: people who use drugs)
Prototyping and rapid iteration	“Low-fidelity” version of a product that contains key functions of interest in order to test a concept, facilitate rapid evaluation and feedback, or answer a specific question (e.g., deciding between two design alternatives). Later, fully functional “high-fidelity” prototypes may be created that are more similar to the final product and typically offer real interactive content	“Low-fidelity”: design sprints, mockups & beta version of sandbox, usability interviews; iterative feedback from community advisory board members “High-fidelity”: pilot version across harm reduction organizations in four counties
Design simplification (of existing intervention parameters and procedures to promote uptake)	Simplification is an overarching principle with specific applications to multiple design activities, such as the processes of scoping product functions and features (i.e., avoid unnecessary options) or determining the ways products present information to users The goal of simplification can either be achieved by (a) keeping primary tasks unchanged, but incorporating new supportive infrastructure or external memory devices to supplement human perceptual abilities (e.g., dashboard instruments that communicate the state of the object in question [such as an automobile]), or (b) reducing the complexity of a task itself (e.g., introducing Velcro to replace shoelaces, or digital watches to replace analog)	Goal A: achieved by tech expert techniques through usability, layout, ease of use, etc. but the primary tasks are unchanged: report an overdose (then having the dashboard with data trends, pulling, hot spots, etc.)
Consideration of system constraints to ensure the end product fits the needs of the targeted end user	Within the context of design, environmental constraints represent properties of an intended destination setting that limit the ways a product will be designed or used. Product design depends largely on this type of constraint, which may include limitations on or requirements for a product's form, function, budget, operating conditions, or time to completion, among others	Process mapping and field observations of harm reduction organization work flow within the context of mobile van outreach and street outreach for homeless encampments

Our approach to optimizing usability was through community-engaged research methods. Community-engaged research is an approach that actively seeks to include and elevate community-based organization perspectives in research [20]. These methods seek to form a partnership between academic institutions and community organizations and are critical to the success of health promotion efforts for stigmatized conditions and vulnerable populations such as overdose and substance use disorder [21]. Our community engaged approach to the UCD phase that focuses on identifying users, user needs, and constraints on use was through our Community Advisory Board (CAB) sessions and individual interviews with key stakeholders. First, we established CABs across 4 pilot sites in Texas that spanned rural, urban and border counties. CAB members were comprised primarily of formal and informal (community led) harm reduction organization leaders, and also included other representatives of community agencies engaged in the field such as first responders and treatment providers. CABs met every two months for two years and were provided with project updates, queried on specific aspects of platform development that emerged over time (e.g., language translation; location tracking) and asked for help recruiting additional CAB members and qualitative interview participants. The rapid, iterative prototyping and simplification phase occurred in our Design Sprint and User Interviews, in which potential users engaged with engineers throughout the development process.

*Qualitative interviews with key stakeholders*. We conducted qualitative interviews among a series of *N* = 44 key stakeholders: 20 harm reductionists and 24 people who use drugs across our four pilot counties in Texas. For this study, we defined harm reductionists as a person who is currently employed or volunteers at a community-based harm reduction organization in Texas. These interviews lasted anywhere between 60 and 90 min and participants were compensated $30 for their time. In these interviews we addressed the following research questions related to both fatal and non-fatal overdoses: (1) What are perceived barriers to overdose reporting among harm reduction organizations?; (2) What are perceived facilitators to overdose reporting among harm reduction organizations?; and (3) What are perceived solutions to improve tracking of opioid-related variables among harm reduction organizations? A semi-structured interview guide as well as a debriefing guide were created specifically for this project. The interview guide contained a combination of structured, open-ended questions and follow-up probes addressing several areas of interest, such as history of personal drug overdose (if applicable), witnessed fatal and non-fatal drug overdose experience/s, overdose prevention and treatment information and resources, thoughts on the overdose reporting platform developed by the project, and ways to motivate people to report overdose through the digital platform.

### Participants

*Eligibility.* The inclusion criteria for harm reduction organization members included: (1) eighteen years or older; (2) volunteer or paid staff member for a harm reduction organization in one of the target counties; and (3) ability to read and speak in English. The inclusion criteria for people who use drugs (PWUD) included: (1) eighteen years or old; (2) used opioids or stimulants in the past 3 months, (3) resides in Texas, and (4) ability to read and speak in English. The exclusion criteria for all participants included: the inability or unwillingness to provide consent, being actively intoxicated, suicidal, or psychotic.

*Recruitment.* Potential participants were screened with a short survey over the phone or via email. The research team coordinated the screening process. If a potential participant fit the inclusion criteria, informed consent was provided and consent was obtained. Participants were assigned a unique ID number and all personal identifying information was removed. Recruitment methods involved flyers, e-mails, telephone, snowball sampling, social media advertising, web-posting, word of mouth and using our Community Advisory Boards (CABs) in each pilot county to help us recruit. In total, we recruited and interviewed 20 harm reduction workers and 24 people who use drugs before reaching data saturation.

### Data collection

Qualitative interviews were conducted by videoconference with two trained researcher staff. One researcher led the interview while the other co-facilitated and took notes. After the interview was completed, the audio recordings were transcribed verbatim by a professional transcription agency. Transcripts were cleaned and scrubbed of all personal identifying information, once returned the cleaned transcripts and the debriefing guides were used for analysis.

### Data analysis

Interview data were analyzed using applied thematic analysis and triangulated to inform development of the harm reduction overdose reporting digital platform. Data from the qualitative interviews were analyzed using applied thematic analysis which was selected for its flexibility and systematic approach in analyzing text-based qualitative data while planning as well as preparing for the data collection [22]. Originally, the team identified emergent themes based on the a priori research goals. Based on these major themes, a working codebook was generated and framework matrix. The designed codebook covered several specific units of data: overdose, drug overdose prevalence, overdose reporting, nontraditional reporting mechanisms, solutions to improve reporting, digital platform structure, digital platform implementation, marketing/branding, and stigma.

Data analysis involved six trained coders (two clinical research associates and four research assistants) by means of a reflexive analysis approach. Each transcript was assigned to two members of the team to code independently using the codebook. Once both members completed their coding process, the members met, while using the reflexive team approach to resolve inconsistencies in coding until they reached a consensus and finalized the coded transcript. The codebook went through multiple iterations due to emerging data in the transcripts that were relative to our research objectives. Additionally, the framework matrix was created to organize the data collected via the interviews by using the debriefing guides and cleaned transcripts to identify developing themes as well as listing direct quotations from the interviews. After the data reached saturation, meaning no new information was detected in our framework matrix or coded transcripts, the team gathered the emergent themes directly related to the digital platform from the coded transcripts, and the framework matrix. There were consistencies identified throughout all data sets in terms of: high priority technical needs, what the user-interface should encompass as well as branding and implementation strategies which will be discussed further in the results section.

### Technology development design sprint

**Fig. 1:**
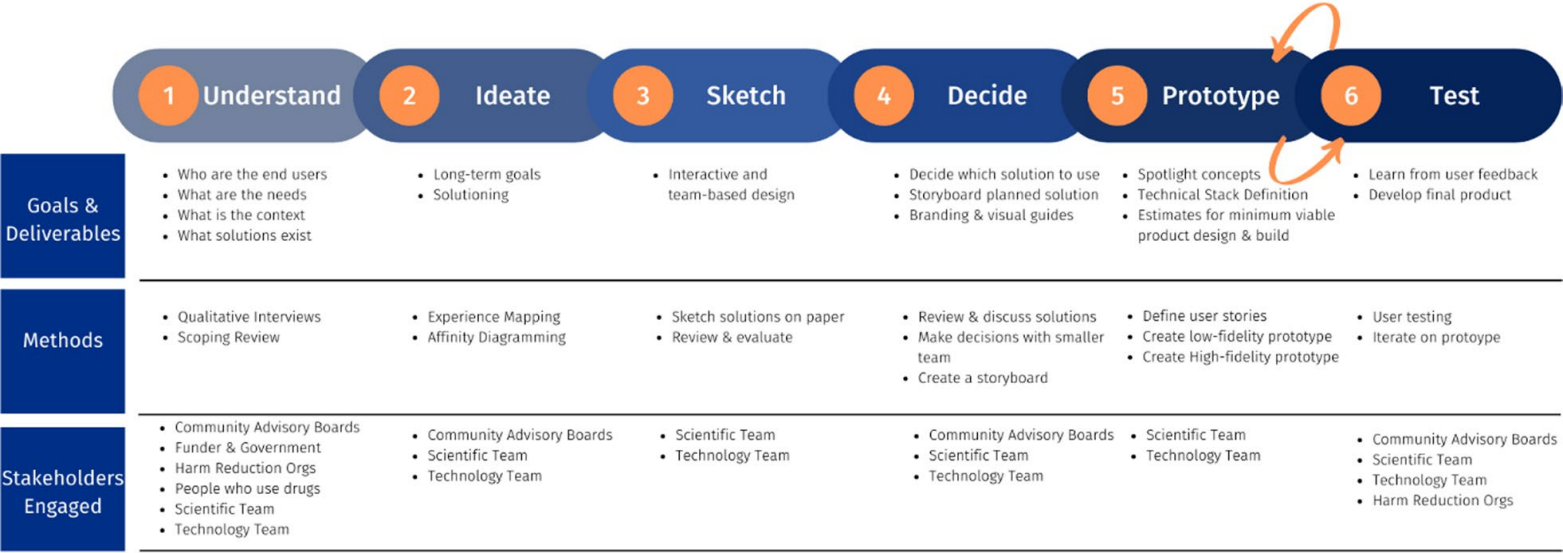
Design sprint method

UCD and community-engaged research principles were again infused throughout the design sprint as community partners were engaged in providing feedback directly to the technical development team on design elements during this technical prototype development phase (see Fig. 1). The design sprint included brainstorming general ideas for the platform, sketching the visuals, mapping out the user experience flow for both people who use drugs and harm reductionist organizations, review of qualitative data review and creating wireframes of the digital platform. Working groups were held with academic, industry, and community partners to decide the optimal technical architecture and user-interface of the overdose platform, and a preliminary implementation strategy.

## Results

### Participant characteristics

A total of 44 participants (*n* = 20 harm reductionists; *n* = 24 people who use drugs) were enrolled in this study. See Table 2 for participant demographics.

**Table 2:** Participant characteristics

	Harm reductionists (*n* = 20)		People who use drugs (*n* = 24)	
	*n*	%	*n*	%
Age				
Median (IQR)	35	(29–50)	36	(29–43)
Gender at birth				
Female	10	50	13	54.2
Male	10	50	11	45.8
Intersex	–	–	–	–
Gender identity				
Male	9	45	13	54.2
Female	9	45	11	45.8
Transgender (FTM)	1	5	–	–
Transgender (MTF)	–	–	–	–
Non-binary	1	5	–	–
Other	–	–	–	–
Unknown	–	–	–	–
Prefer not to answer	–	–	–	–
Sexual orientation				
Gay/Lesbian	2	10	1	4.2
Straight/Heterosexual	11	55	21	87.5
Bisexual	5	25	2	8.3
Other	3	15	–	–
Prefer not to answer	–	–	–	–
Race				
Black/African American	2	10	1	4.2
White/Caucasian	14	70	20	83.3
Asian	2	10	–	–
American Indian	1	5	–	–
Pacific Islander/Native Hawaiian	–	–	–	–
Other	4	20	5	20.8
Ethnicity				
Hispanic or Latino	8	40	10	41.7
Not Hispanic or Latino	11	55	13	54.2
Prefer not to answer	1	5	1	4.2

Thematic analysis of the qualitative interviews with key community stakeholders in harm reduction (HR) and people who use drugs (PWUD) produced critical information regarding high priority technical solutions and needs for the harm reduction organization version of the digital platform’s design, structure, and features. Additionally, barriers and facilitators that would affect adoption and usage of the platform within harm reduction settings were expressed. The data regarding these perceived solutions, barriers and facilitators were centered primarily around data collection, data reports, and user interface (UI) design.

### Perceived barriers and facilitators related to a digital overdose reporting platform usage within harm reduction organizations

*Data collection barriers and facilitators.* Participants expressed a crucial need for anonymity and security of personal information entered into the platform, emphasizing that this would be one of the most necessary facilitators for platform use. Harm reduction stakeholders repeatedly noted the critical importance of the established trust between themselves and their clients in the community, whose data the platform largely aims to collect. A technology innovation must prioritize maintaining this trust in order to be effective. One participant said, "*I would make use of an app, especially if it allowed [reporting overdoses] to be more anonymous. In my past, that was the only thing that kept me from reaching out*" (PWUD, Pt. 129). Ensuring the overdose reporting platform does not break relationships through the risk of user identification was described as crucial due to fear of legal repercussions or being tracked by law enforcement, particularly in Texas. Additionally, while collection of demographic data was described as an important data metric and as a solution to overdose surveillance accuracy, the challenge of collecting these data from participants was described as a potential barrier. One participant said that collecting demographics such as date of birth was “*kind of a touchy subject when it comes to confidentiality*” for clients (HR, Pt. 139). This is likely due to fear of stigma and mistrust in healthcare and criminal justice institutions among this vulnerable population. These data illustrate the need to be selective with which demographic data points to include in the platform in a comprehensive system on a need-to-know basis and the need to limit access to these data among institutions that may abuse this information.

Including community members in the collection of opioid-related data through the harm reduction platform was considered another strong facilitator to trust and adoption. Participants noted that including non-traditional first responders, such as community gatekeepers, witnesses, or people who experience an overdose, could make them feel empowered and even more trusting in the platform, saying “*any kind of most updated iOS or Android type of application we can create, and it's easy to download to somebody's phone, allow participants the safety to report on their own—I feel like that should be the goal, to empower our participants to be able to report that information when needed and as they feel comfortable*” (HR, Pt. 116). This idea could also have significant effects on the accuracy and frequency of overdose reporting among communities, as participants noted that "*if people started having more positive experiences when they did report it, then word would get around what really happens when you report it. Like, that's how it happens when you report an overdose. They don't frisk everybody there and threaten to throw them in jail*" (PWUD, Pt. 135). By encouraging non-traditional first responders to be active and report overdoses, data accuracy could improve through community reporting.

*Data report barriers and facilitators.* Regarding data reports, HR stakeholders explained the need to generate reports to receive funding as primarily grant-funded organizations, so the ability to easily pull reports straight from the platform was a strong facilitator for adoption and championing at the administrative level of the organization. The expressed benefits to using data reports, the successive aftermath of improving exposure and access to resources and services among communities, and the ability to provide strong reports for funding applications feature all support the critical need for generating robust data reports among harm reduction organizations.

*User interface design barriers and facilitators.* Harm reduction organizations requested a quick and flexible design not only to assist with seamless data entry, but also to preserve positive rapport and interrupt client interaction as minimally as possible. The design needed to be conducive to street outreach and having conversations with PWUD. Participants described the impersonal effects of lengthy data collection processes, noting that “*it’s horrible if you’re trying to take someone’s time to get data, and then they’re just waiting for things to load*” (HR, Pt. 163). Though the direct benefit of this recommendation affects their clients, painless data entry methods would ultimately be seen as a facilitator for HR users since preserving these community relationships and face-to-face interaction with their clients was integral to their workflow standards.

In general, data collection and entry were seen as barriers to adoption and use of the platform while serving clients, "*especially with individuals who are using substances, if you want them to trust you and feel comfortable talking to you, if you pull out a paper and you’re writing stuff down, or a tablet or something, sometimes that really turns people off*" (HR, Pt. 140). To account for this, participants suggested incorporating a feature allowing outreach workers to submit a report form electronically after an interaction or conversation (HR, Pt. 105). This feature was mentioned throughout interviews as a facilitator to preserving these crucial face-to-face connections and relationships. Participants noted that building an offline mode and save feature would facilitate this type of interaction.

The ability to provide clients with community resources was a frequent theme and a significant facilitator in adoption and sustainability of trust in the platform. Resource connection was described as a large part of harm reduction services, so participants expressed significant interest in using the platform as a centralized collection of shareable resources, with printable options available straight from the platform.

### Perceived solutions to improve tracking of opioid-related variables among harm reduction organizations

*Data collection solutions.* The importance of collecting minimal personal client data regarding overdose events arose frequently throughout the interviews. Harm reductionists (HR) acknowledged the importance of using demographic data to provide robust and accurate drug use data trends, with one participant noting that collecting “*personal [identifying] information so it [could] be compared throughout the database to see if it's been collected already*” was a potential solution to data entry duplication (HR, Pt. 139). Additionally, many participants reported that they already collect basic demographic data in their current organizational tracking processes, which was described as "*absolutely important and vital*" information to collect (HR, Pt. 120). Participants encouraged demographic data collection and learning about this solution was significant when conceptualizing the platform design, as participants had consistently commented on the importance of only obtaining the minimal data necessary from participants in these sensitive conversations.

Another significant need for HR stakeholders regarding data collection was to make the platform universal and interoperable with existing systems already collecting opioid overdose-related data. Participants described potential organizations to include whose systems could connect with the platform: "*I wish there was a central reporting system where hospitals, first responders, law enforcement, medical examiner, people…were providing this kind of information*" (HR, Pt. 140) to give a complete and accurate view of overdose event occurrences. Not only did participants suggest interoperability to prevent data duplication, but also to be inclusive of non-traditional first responders, which was seen as a powerful solution to fill the gap in data for people who do not call 911 in an overdose event. One participant said, “*I wanna say more than 90 percent of overdoses that we hear about [in harm reduction] were never called 911 for. So that's something I hear about sometimes from people when I talk about tracking this information. They're like, ‘Oh, I'm just gonna pull EMS EHR [electronic health records],’ and, like, that's nice. You're probably capturing a tiny, little bit of it. It's probably a very specific subset of the population that feels confident enough to call 911*” (HR, Pt. 101). By designing the platform to be integrated into existing harm reduction organizational systems, as well as to be user friendly for nontraditional first responders, participants felt that both duplicated and missing data points could be addressed while establishing trust within the community they serve.

*Data report solutions.* Participants expressed that the ability to access and use their own harm reduction organization’s data from the platform for increased insight on local community overdose activity was also a significant solution to improve opioid trend tracking. Stakeholders were interested in the ability to use real-time reports to alert communities of trends and spikes in overdoses and to quickly identify clusters by location to focus overdose prevention efforts. One participant suggested "*at least being able to see if there are neighborhood clusters. Or a particular area. I mean that would be incredibly helpful for our services. If we could see that a bunch of people overdosed even if we didn't know from what. If there is a spike in the map, we—and other organizations—could figure out what's going on in that community*" (HR, Pt. 105). Additionally, participants saw benefit from having these data to inform what areas need specific community resources. Some participants suggested that these data could improve resources and services simply by giving them more exposure through the platform, as "*there are tons of resources that people just don't know about*" (PWUD, Pt. 136). Participants were asked to describe a solution to attain these organizational data, and a common theme outlined the need for a harm reduction login to track metrics within and beyond their organization. This solution would be a first step to differentiate overdose data variables recorded in their organization versus those submitted by community members, allowing better tracking of the overdose-related trends seen within their respective organizations as well as their communities.

*User interface design solutions.* As a unique user group, participant data highlighted the need for a customized UI to meet harm reduction organizational needs, including potential users who could interact with the platform and the importance of maintaining positive relationships with clients. One consistent theme across interviews to account for these characteristics was the need for a simple, user-friendly, and quick design to account for HR volunteers and clients who may use the platform. Participants noted various reasons that the overdose reporting platform should be "*very practical and easy to use*,” stating that "*if different people are going to be using it you have to make it really easy to understand because then you get misclassification of information*" (HR, Pt. 141, 106). Additionally, some clients “*might get fatigued. As it is, they’re already using [drugs], so their patience is not too good*” (HR, Pt. 141). Participants also noted that “*the general population are at the 6th grade reading level*” (HR, Pt. 119), so designing the interface as user-friendly and simple is vital for these potential users.

Various other recommendations for platform design resulted from qualitative interviews. Some of the high-priority features included multilingual options, broader data collection on type of drug involved in overdose events, and community resources being readily available to disseminate to clients. As Texas contains cities bordering Mexico, many participants highlighted the need for a multi-lingual interface; Spanish was the primary language requested in addition to Vietnamese. Additionally, stakeholders felt it was important to be inclusive in drug type and type of overdose (fatal or nonfatal). Many HRs interviewed were made regularly aware of drug types and trends among their local communities and expressed a need to prioritize tracking opioid and stimulant overdoses, as well as to include other substance types in the platform to account for the rising trends in poly-substance use and to avoid exclusion of other drugs and overdoses.

Providing resources to community members was another high priority need for both HR and PWUD, as both of these groups interact routinely. Suggested resources included video tutorials or wikihows (pictures with steps broken down) on how to properly/safely use drugs, links to Narcotics Anonymous or Alcoholics Anonymous meetings and needle exchange schedules, methadone clinics and treatment centers (PWUD, Pt. 134) as well as overdose hotspots, emergency-style rehab or detox centers for immediate support, and support groups (PWUD, Pt. 135).

### Harm reduction organization dashboard prototype

Formative research informed the development of the prototype for the harm reduction organization dashboard. We engaged the community advisory boards across four diverse counties throughout the entire design process to co-design the dashboard prototype. We conducted user-experience testing throughout the design sprint process to ensure key features were included and the dashboard was acceptable and useful among staff, volunteers, and administration within the harm reduction organizations. This process was critical to establish trust within the community among the target population of people who use drugs.

**Fig. 2:**
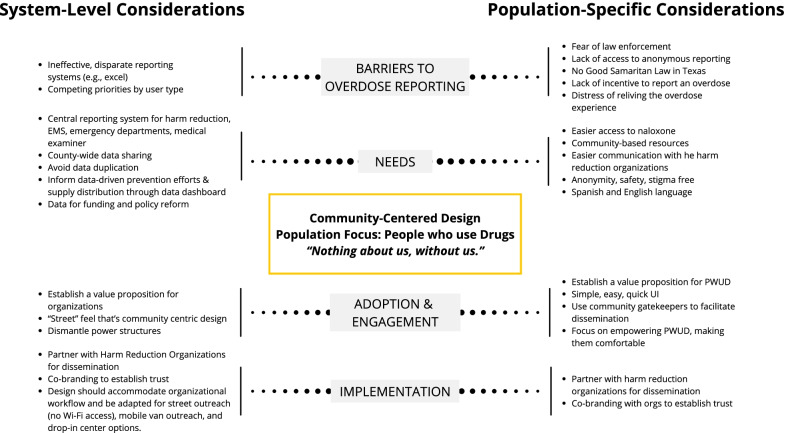
Emergent themes

An important component of the dashboard design included a warm, colorful user interface that avoids a “clinical” or “healthcare” connotation and had more of a “street feel”. Further, the dashboard should not acknowledge any government or higher education entity; instead it should be fully community-driven in branding. Key features of the dashboard include an overdose reporting form, an organization-specific dashboard with log-in capabilities, and a Spanish/English toggle. The organizational dashboard allows customization and tracking of harm reduction supplies, data analytics pertaining to overdose reporting metrics, and a high-level overview of the organization’s impact through community outreach activities (see Fig. 2).

## Discussion

This study highlighted a community-engaged, user-centered design methodology to inform the development of a digital platform to improve overdose surveillance and data-driven prevention efforts among harm reduction organizations in Texas. Harm reduction organizations are embedded within the community, have established trust and heightened access to individuals at high risk for overdose. Further, these organizations traditionally are under-resourced and provide necessary community outreach to some of the most vulnerable and underserved people who use drugs. They are a conduit to healthcare access among a population that experiences significant health inequity in accessing the healthcare system. Many individuals who experience overdose do not contact EMS or interact with the healthcare system due to stigma and fear of law enforcement [23]. This is particularly true within the context of Texas where the Good Samaritan law does not sufficiently protect individuals who report and experience an overdose from legal repercussions. A Good Samaritan law has been passed in 41 states in the USA in response to the opioid crisis [24]. These laws aim to encourage people who use drugs to seek medical care following an overdose through extending legal immunity from arrest, charge or prosecution for certain controlled substance possession and paraphernalia offenses. This immunity extends to the person who experiences an overdose and bystanders who call 911.

Existing data analytics for overdose rely on data from emergency management services (EMS), emergency departments, and death records to calculate public health statistics. Consequently, only individuals who encounter the health care system following an overdose are recorded within these statistics. Further, these data only reflect fatal overdoses. Capturing overdose data among populations who do not access these systems is critical. Overdose surveillance technologies tailored to harm reduction organizations may be an effective avenue to collecting overdose data as these organizations have established trust in the community among people who use drugs and frequently interact with people who have experienced or witnessed an overdose. Many of these organizations currently collect data related to overdose incidents for their organizational records; however, no system exists to aggregate these data to inform community response efforts. Brandeis University’s Opioid Policy Research Collaborative has recently launched a drug check mobile-based application called STREETCHECK which streamlines community illicit drug sample collection and communicates results back to people who use drugs. This community-academic partnership is a strong example of developing harm reduction-oriented digital platforms that prioritizes the needs of people who use drugs and facilitates harm reduction efforts through community-based harm reduction organizations.

Further, creating overdose data collection tools designed for people who use drugs that provide an added benefit or incentive for reporting may be another effective pathway to improve data. A recent study found that approximately 77% of people who inject drugs have a mobile phone and of those with a mobile phone, 88% have a smart phone with voice and internet service [25]. Future studies should incorporate people who use drugs in a co-design process to solve this problem and develop a solution that preserves trust, protects the population, and improves overdose data. Public health efforts will benefit from improved analytics via the inclusion of non-fatal overdoses within surveillance efforts. A robust digital ecosystem that combines traditional overdose surveillance methods collected through EMS records, death certificates, and emergency department records combined with data collected from non-traditional reporters such as harm reduction organizations and people who use drugs are needed. Importantly, technologies aggregating data sources need to develop methods to address data redundancy and quality control. These analytics have the capacity to improve data-driven response efforts, particularly among community-based harm reduction organizations.

Findings from this study should be taken in light of several limitations. Data were collected among harm reduction organizations and PWUD in Texas, as such findings may not generalize across other states and may reflect existing State policies related to harm reduction at the time of data collection. Data collection for this study was conducted throughout the COVID-19 pandemic which severely impacted harm reduction organization operations. Data from this study may reflect perspectives of harm reductionists as a result of the COVID-19 pandemic effects. Findings presented informed the development of a prototype overdose reporting platform for harm reduction organizations. As such, future studies need to evaluate acceptability, usability, and feasibility of this overdose reporting platform within harm reduction organizations.

The use of combined community-engaged research methods with a user-centered design approach informed development of a prototype overdose reporting dashboard for harm reduction organizations. Data gathered from community advisory board meetings, qualitative research methods, and the co-design process will guide ongoing development of the platform. Future evaluation of the platform will evaluate the utility of such technology toward improving overdose surveillance and informing data-driven prevention efforts uniquely tailored to harm reduction organizations serving high-risk people who use drugs.
